# Quantitative neuroanatomy of all Purkinje cells with light sheet microscopy and high-throughput image analysis

**DOI:** 10.3389/fnana.2015.00068

**Published:** 2015-05-27

**Authors:** Ludovico Silvestri, Marco Paciscopi, Paolo Soda, Filippo Biamonte, Giulio Iannello, Paolo Frasconi, Francesco S. Pavone

**Affiliations:** ^1^National Institute of Optics, National Research CouncilSesto Fiorentino, Italy; ^2^European Laboratory for Non-Linear SpectroscopySesto Fiorentino, Italy; ^3^Department of Information Engineering, University of FlorenceFlorence, Italy; ^4^Department of Engineering, University Campus Bio-Medico of RomeRome, Italy; ^5^Institute of Histology and Embryology, Catholic University of the Sacred Heart “A. Gemelli”, RomeItaly; ^6^Department of Physics and Astronomy, University of FlorenceSesto Fiorentino, Italy; ^7^International Center for Computational NeurophotonicsSesto Fiorentino, Italy

**Keywords:** quantitative neuroanatomy, Purkinje cells, cerebellum, light sheet microscopy, image analysis, brain imaging

## Abstract

Characterizing the cytoarchitecture of mammalian central nervous system on a brain-wide scale is becoming a compelling need in neuroscience. For example, realistic modeling of brain activity requires the definition of quantitative features of large neuronal populations in the whole brain. Quantitative anatomical maps will also be crucial to classify the cytoarchtitectonic abnormalities associated with neuronal pathologies in a high reproducible and reliable manner. In this paper, we apply recent advances in optical microscopy and image analysis to characterize the spatial distribution of Purkinje cells (PCs) across the whole cerebellum. Light sheet microscopy was used to image with micron-scale resolution a fixed and cleared cerebellum of an L7-GFP transgenic mouse, in which all PCs are fluorescently labeled. A fast and scalable algorithm for fully automated cell identification was applied on the image to extract the position of all the fluorescent PCs. This vectorized representation of the cell population allows a thorough characterization of the complex three-dimensional distribution of the neurons, highlighting the presence of gaps inside the lamellar organization of PCs, whose density is believed to play a significant role in autism spectrum disorders. Furthermore, clustering analysis of the localized somata permits dividing the whole cerebellum in groups of PCs with high spatial correlation, suggesting new possibilities of anatomical partition. The quantitative approach presented here can be extended to study the distribution of different types of cell in many brain regions and across the whole encephalon, providing a robust base for building realistic computational models of the brain, and for unbiased morphological tissue screening in presence of pathologies and/or drug treatments.

## Introduction

Since the times of Golgi and Ramòn y Cajal, technological advances always played a crucial role in helping neuroanatomists to disentangle the complex architecture of the mammalian brain. Indeed, methodological innovations always brought, sooner or later, to deep biological insights, and to novel paradigms of neuroanatomical investigation. The development and refinement of imaging techniques, like electron microscopy (Gray, 1969), fluorescence optical microscopy (Lichtman and Conchello, 2005) and magnetic resonance imaging (Duyn and Koretsky, 2008), allowed studying brain organization on different scales and resolutions, leading to the definition and the study of different anatomical structures, ranging from single synapses (Palay, 1956) to entire neurons (Meinertzhagen et al., 2009), from cortical columns (Rockland, 2010) to long-range fiber tracts (Tench et al., 2002).

Imaging technology has thus played a crucial role in the development of contemporary neuroscience; anyway, its limitations can somehow distort the great picture of the brain we are painting, providing us with partly biased representations of the central nervous system. One traditional limitation in neuroanatomical reconstructions is the relatively small throughput: the imaged volume decreases as the resolution increases. Thus, for instance, dendritic morphology is usually investigated on the level of few neurons, while cell spatial distribution is analyzed within single cortical columns (or structures of similar size). The loss of resolution when zooming out to large volumes hides to the researcher any possible long-range correlation in the fine details of neuronal organization. Furthermore, since high-resolution studies are limited to small areas, one has usually to select the region of interest in advance, usually based on previous literature, at the risk of neglecting unexpected neuroanatomical features in other parts of the brain. This exposes the analysis and thus the conclusions drawn upon it to potential bias.

Recent advances in imaging technology can help contemporary neuroanatomists to afford a more comprehensive view of the brain cytoarchitecture. Indeed, nowadays there is a clear trend toward high-throughput microscopy methodologies (both optical and electronic), as larger and larger portion of tissue can be imaged with higher and higher resolution (Briggman and Denk, 2006; Osten and Margrie, 2013). On the one hand, focused-ion-beam milling serial electron microscopy (Knott et al., 2008) and multiple-beam scanning electron microscopy (Keller et al., 2014) allows reconstructing small brain regions with nanometric resolution, providing useful data for the reconstruction of local connections. On the other hand, light sheet microscopy (LSM; Dodt et al., 2007; Silvestri et al., 2012), coupled with chemical clearing of the tissue (Becker et al., 2012; Chung et al., 2013; Tomer et al., 2014) can be used to image entire murine brains with micrometric resolution without the need for physical sectioning. Optical methods based on serial sectioning (Li et al., 2010; Ragan et al., 2012; Gong et al., 2013) can as well-provide whole-brain μm-resolution images, although at the cost of destroying the sample.

Anyhow, the impact of all these technical improvements on XXIst century neuroanatomy has been very limited hitherto. In fact, the amount of data produced by novel, high-throughput imaging methods easily falls in the TeraByte range or beyond, moving the throughput bottleneck from data production to data analysis. Large-scale projects have benefit from the massive contribution of human supervision in manual or semi-manual data segmentation tool, either hiring dozens of students (Briggman et al., 2011) or leaning on crowd contributions via an interactive videogame (Kim et al., 2014). Nevertheless, such brute-force approaches are out of the reach for most laboratories worldwide, which have to cope with limited human and financial resources. Automatic methods for management of large images, for their visualization and annotation, and for cell soma localization, have been recently described (Peng et al., 2010, 2014; Bria and Iannello, 2012; Frasconi et al., 2014). Since these automatic methods are conceived to minimize the computational resources needed (most of them can run even on high-end workstations), they offer the possibility for neuroanatomists to finally solve the data bottleneck and reach a real output from high-throughput imaging methodologies.

**FIGURE 1:**
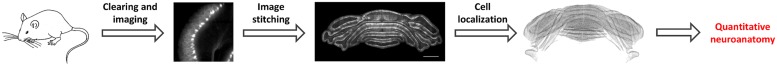
**Experimental pipeline for large-volumes quantitative neuroanatomy**. After animal fixation, the brain is render transparent and imaged with high-throughput light sheet microscopy. Raw image stacks are then stitched together, and a software for automatic cell localization applied. The resulting cloud of points representing the position of labeled cells can be the starting point for many different quantitative neuroanatomical analysis.

Here, we present a complete experimental pipeline, integrating recent innovations in the fields of imaging technology and computer science, to extract quantitative information about the three-dimensional distribution of a selected neuronal population. The approach we describe, summarized in **Figure 1**, encompasses specimen clearing with organic solvents, imaging with confocal LSM, image stitching and automatic soma detection, and eventually allows localizing each individual fluorescent neuronal soma across a large brain region. We demonstrate this pipeline by reconstructing the full neuroanatomy of the Purkinje cells (PCs) layer which are known to be the most important inhibitory neurons that carry the only output of the cerebellar cortex. To this aim, we imaged the whole cerebellum of a B6C3Fe-L7-EGFP (L7-GFP) mouse (Oberdick et al., 1990). Starting from the point cloud representing the position of single PCs, we are able to clusterize the PCs in groups based on their representation in the 3D space, which might delineate some significant neuroanatomical parcellation and reveal isolated neurons. Finally, by locally unwrapping the two-dimensional lamellar structure of the Purkinje layer, we can locate empty spaces within the layer, known as gaps. Such gaps are thought to play a significant role in autism spectrum disorders (McKimm et al., 2014). With the high-output comprehensive approach described here, we are able to draw a map of Purkinje gaps in the whole cerebellum and highlight their spatial organization, which would be hardly accessible with conventional techniques.

## Materials and Methods

### Sample Preparation

The whole cerebellum from a young male L7-GFP mouse was cleared following a protocol based on the one reported by Dodt et al. (2007). A post-natal day (PND) 10 mouse was deeply anesthetized by hypothermia and intraperitoneal injection of tribromoethanol (220 mg/kg), and transcardially perfused with 0.1 M PBS (pH 7.4) followed by 4% paraformaldehyde (PFA) in 0.1 M PBS for fixation. The brain was then removed from the skull, post-fixed in 4% PFA overnight at 4∘C and stored in PBS at 4∘C. Afterward, the cerebellum was dissected out and embedded in a 0.5% (w/w) low melting point agarose gel, prepared in 10 mM sodium borate buffer (pH 8). The embedded sample was dehydrated in a graded ethanol series (30, 50, 80, 96% 2 h each, 100% overnight). The ethanol was diluted in sodium borate buffer to avoid considerable pH variations that would possibly decrease EGFP fluorescence. After dehydration, we incubated the specimens in freshly prepared clearing solution (Benzyl Alcohol/Benzyl Benzoate 1:2, BABB) for ∼36 h. All dehydration/clearing steps were performed at room temperature (18–22∘C). The dehydration and clearing procedure led to a linear shrinkage of the tissue of about 25% (Silvestri et al., 2014), so the volume is reduced to about 42% of its original size.

All experimental protocols involving animals were designed in accordance with the regulations of the Italian Ministry of Health.

### Confocal Light Sheet Microscopy

In LSM, the sample is illuminated with a thin sheet of light, confining fluorescence excitation in the focal plane of detection optics (Keller and Dodt, 2012). In this way, optical sectioning (i.e., 3-dimensional resolution) is afforded in a wide-field detection scheme, where millions of pixels are collected simultaneously by a camera instead of sequentially as in point-scanning techniques (as standard confocal or two-photon microscopy). This high imaging throughput with high 3-dimensional resolution makes LSM an ideal technique to reconstruct the anatomy of large specimens, as murine brains.

The custom made confocal LSM used here has been described in detail in Silvestri et al. (2012). Briefly, planar illumination is achieved by fast scanning of a line inside the specimen (Keller et al., 2008), and a de-scanning system in the detection path is used to create a fixed image of the scanning excitation line. At the position of this fixed image a linear spatial filter (slit) is used to block out-of-focus and scattered light. This confocal line detection affords a contrast enhancement of 100% in cleared specimens (Silvestri et al., 2012). A third scanning system re-creates a 2-dimensional image onto the chip of an electron-multiplying charge-coupled device (EM-CCD), which collects the photons producing a digital image. The objective used to collect the data analyzed here was a Nikon Plan SLWD 20 × (NA 0.35), with long working distance (24 mm) and designed to work in air; slit width was set to 1 μm (in object space). During imaging, the specimen was kept immersed in clearing solution inside a custom chamber. The chamber was mounted on a XYZθ stage, assembled using three linear stages and a rotation one (M-122.2DD and M-116.DG, Physik Instrumente, Germany).

To collect a full optical tomography of the cerebellum, many parallel adjacent image stacks were acquired to cover the entire volume. A partial overlap of about 10% of the field of view was introduced to allow subsequent stitching of image tiles (see below). A custom-made software written in LabVIEW (National Instruments, Austin, TX, USA) orchestrated all the hardware components of the microscope to perform automated imaging of the volume.

### Image Stitching

To stitch the tiled raw image, the TeraStitcher tool (Bria and Iannello, 2012) has been used. TeraStitcher is a free and fully automated 3D stitching tool specifically designed to match the special requirements coming out of teravoxel-sized tiled microscopy images. It is able to stitch such images in a reasonable time even on machines with limited resources. It can be freely downloaded from https://github.com/abria/TeraStitcher.

TeraStitcher consists of a pipeline of six stages. After the import stage, in which the raw data are scanned to reconstruct the nominal position of every tile in the instrument space, the alignment between every pair of adjacent tile is computed. The alignment stage is based on the MIP-NCC algorithm, i.e., an alignment strategy based on using 2D Normalized Cross-Correlation on Maximum Intensity Projections in each direction of the two volumes to be aligned. MIP-NCC is applied to multiple homologous sub-stacks of any pair of adjacent tiles, so as multiple alignments for each tiles pair are computed. An index measuring the reliability of each alignment is also computed and used in the third stage to select the most reliable alignment for each tiles pair. In the fourth stage, alignments between tiles pairs with reliability below a given threshold are discarded. Indeed, these alignments should not affect final image stitching, since they correspond with high probability to adjacent tiles that share only empty sub-volumes. In the fifth stage, the remaining reliable and possible redundant alignments are given as an input to an optimization algorithm that uses alignment reliabilities to find the tile positions that minimizes the global alignment error. Finally, in the last stage, according to the output alignments of the fifth stage, overlapping tiles are merged, and the final stitched image generated.

It is worth noting that the above strategy is computationally cheap, greatly limits memory occupancy, and requires only two reads and one write of the whole image data. The size of the raw data corresponding to the whole mouse cerebellum was 198,78 Gbytes. On a workstation with 96 GB of RAM, 9 TB of disk space, 2 quad-core CPUs at 2.26 GHz, TeraStitcher took 771 min to stitch the whole volume, 539 of which spent in I/O operations. The peak memory occupancy was only 1.13 Gbyte.

### Automatic Cell Localization

We proceed in separate stages to identify the 3D coordinates all Purkinje somata in the cerebellum image. The method is detailed in Frasconi et al. (2014) and briefly summarized here. Since the image is large (1.2 × 10^11^ voxels), it is not practical to process it as a whole. We therefore split it into 9000 overlapping substacks of size 280 × 282 × 246. This approach has other advantages, including the ability to exploit data parallelism in a computer cluster.

The first stage of the identification pipeline is called *semantic deconvolution* and aims at addressing the high variability in quality and contrast found in confocal LSM images. Semantic deconvolution is performed by training a deep neural network to clean-up small (13 × 13 × 13 voxels) image patches so that cell somata ideally appear as small white spheres in a black background. The neural network has an input layer of 2197 units, two hidden layers of 500 and 200 sigmoidal units, respectively, and a linear output layer of 2197 units that are supervised with a clean version of the input image patch. The network is run in a convolutional fashion throughout whole substacks, using a stride of 4 voxels.

**FIGURE 2:**
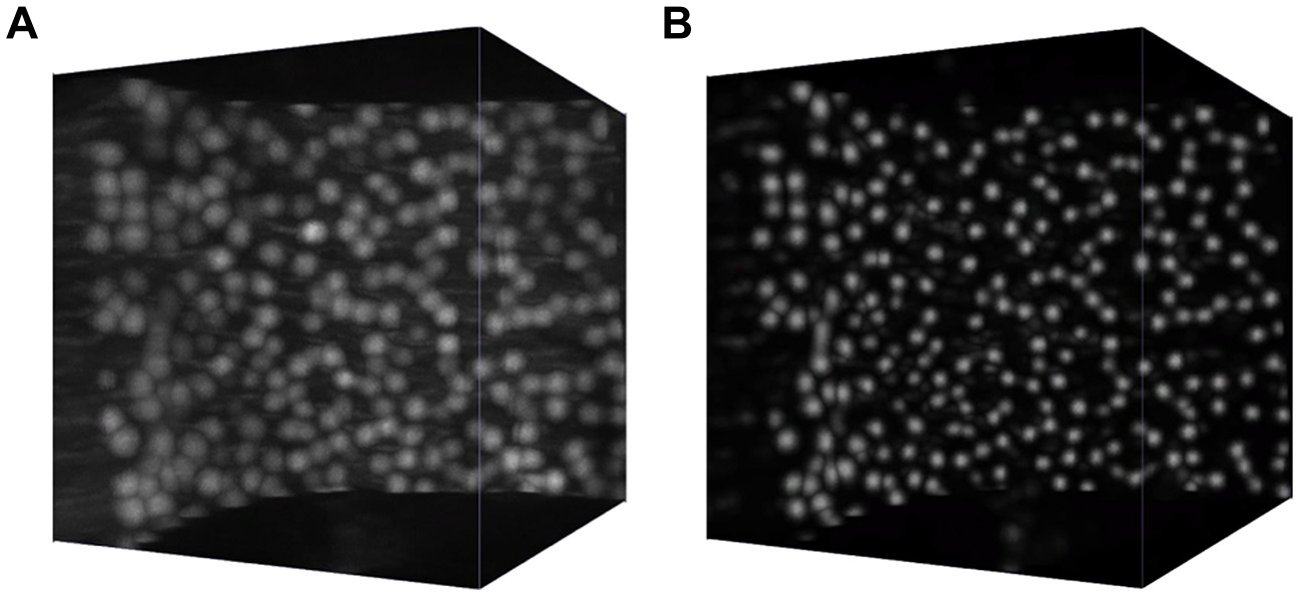
**Semantic deconvolution**. A small volume from the cerebellum of an L7-GFP mouse before **(A)** and after **(B)** semantic deconvolution. On the final image is much easier to run a reliable automatic localization algorithm.

Such an approach allows us to obtain a significant speedup over the naive approach where a network with a single output was trained to predict the conditional probability that the central voxel of the patch belongs to a cell soma. Processing the whole cerebellum image takes about 2 days on a small cluster with 32 Xeon cores (compared to an estimated 2 months running time for the naive approach). The effect of semantic deconvolution is illustrated in **Figure 2**.

The second stage identifies somata coordinates using a variant of the mean shift algorithm (Comaniciu and Meer, 2002) a non-parametric clustering algorithm that takes as input a data set of points L (described in our case by their 3D coordinates *x*, *y*, *z*, and the corresponding gray-level intensity), and a set of seed points *S*. It partitions *L* into *k* subsets, each representing the voxels in a given soma. More precisely, *L* contains all foreground voxels in a given substack, where the foreground threshold *t* is determined by a two-levels maximum entropy algorithm (Sahoo et al., 1988). A voxel is included in the seed set *S* if the following two conditions hold true simultaneously: (1) the voxel is a local maximum in the 3D image, and (2) the average intensity in a sphere of radius r around the voxels is above the foreground threshold *t*. Subsequently, for each seed *s* in *S*, the mean shift algorithm iterates the following two steps until convergence: (1) place a spherical kernel or radius *R* around *s* and compute the center of mass of the points falling within the kernel (using voxel intensities as the “masses”); (2) replace *s* by the center of mass. Overall, the two parameters controlling the algorithm behavior are the radius *r* of the seed ball and the radius *R* of the mean shift kernel. Both are interpretable in terms of geometrical properties of the image contents and, in facts, best results tend to be obtained when *r* and *R* are set to values close to the expected Purkinje radius (6 voxels at the micron image resolution used in this study). Note, however, that smaller values of *r* favor higher recall^1^ (at the expense of precision).

The third stage aims at further reducing the false positive rate by exploiting domain knowledge about the cerebellum cytoarchitecture. In particular, the cerebellum cortex folds into folia that can be modeled as two-dimensional manifolds. As it turns out, most of the false positives detected by mean shift actually correspond to fragments of axon bundles or various other fragments of neurites where GFP was expressed. Since these false detections are very often found far away from the Purkinje layer the precision can be significantly improved by estimating the distance between any predicted soma center and the manifold formed by the nearby predicted centers. For this purpose, we used a combination of manifold learning (via the Isomap algorithm Tenenbaum et al., 2000) and locally weighted regression (Cleveland and Devlin, 1988). Significant improvements can be obtained by discarding all predictions whose estimated manifold distance exceed a certain threshold.

The software for automatic cell localization, and the results shown in this paper, can be downloaded from http://bcfind.dinfo.unifi.it/. Developers can found extensive documentation on the same website.

### Cell Clustering

The algorithm used to compute the clusters of connected somas from the cell cloud uses the notion of k-nearest neighbors (kNNs; Cover and Hart, 1967) and it works as follows. Given two cells c_i_ and c_j_ we introduce the relation:

$$c_{i}\overset{k}{↔}c_{j}$$

If c_i_ ∈kNN(c_j_) ∧c_j_ ∈kNN(c_i_), the connected clusters are the equivalence classes of the transitive closure of relation $\overset{k}{↔}$.

In practice, for each cell c_i_ (with *i* = 1, 2, …, *m*) of the cloud, the algorithm searches its kNNs somas that are collected in the set $s_{i}=\left\{c_{j}^{\left(i\right)}\right\}$, with *j* = 0, 1, …, *p* and *p* ≤*k*. Next, the algorithm runs again a kNNs search for each element of s_i_ in order to determine if $c_{j}^{\left(i\right)}$ is a neighbor of c_i_. If this is the case, the somas c_i_ and $c_{j}^{\left(i\right)}$ are correlated and assigned to the same cluster n_n_. Now, the aforementioned operations are repeated for all cells assigned to n_n_, i.e., the algorithm tries to expand the net n_n_ looking for new connections. The growing phase of the cluster n_n_ ends when the kNNs search return an empty set or a set which members are all already in n_n_, and the algorithm picks a new cell c_i_ (which does not belong to any n_n_ estimated so far) from the cloud and executes again all the previous computations. Note that, when a cell c_i_ does not belong to any cluster, i.e., it is not in the set s_i_ of any of its kNNs, c_i_ is said to be an *isolated* cell.

The result of this algorithm is a list of clusters composed of somas that are mutually kNN-connected in the space and a list of isolated cells. In general, k is chosen to be a small integer. Indeed, small values of k fragment the cloud of somas in relatively small clusters. Conversely, due to the observed spatial distribution of PCs, the higher the value of k, the larger the number of cells assigned to the same cluster. Note also that clustering with higher k values is less sensitive to possible errors in cell localization. Multiple analysis with different values of k may highlight the presence of clearly defined and stable neuronal clusters, which might have a direct neuroanatomical significance.

The software used for cell clustering can be downloaded from https://bitbucket.org/paolosoda/manifold-cluster-cell. Developers can found extensive documentation on the same website.

### Gap Localization

**FIGURE 3:**
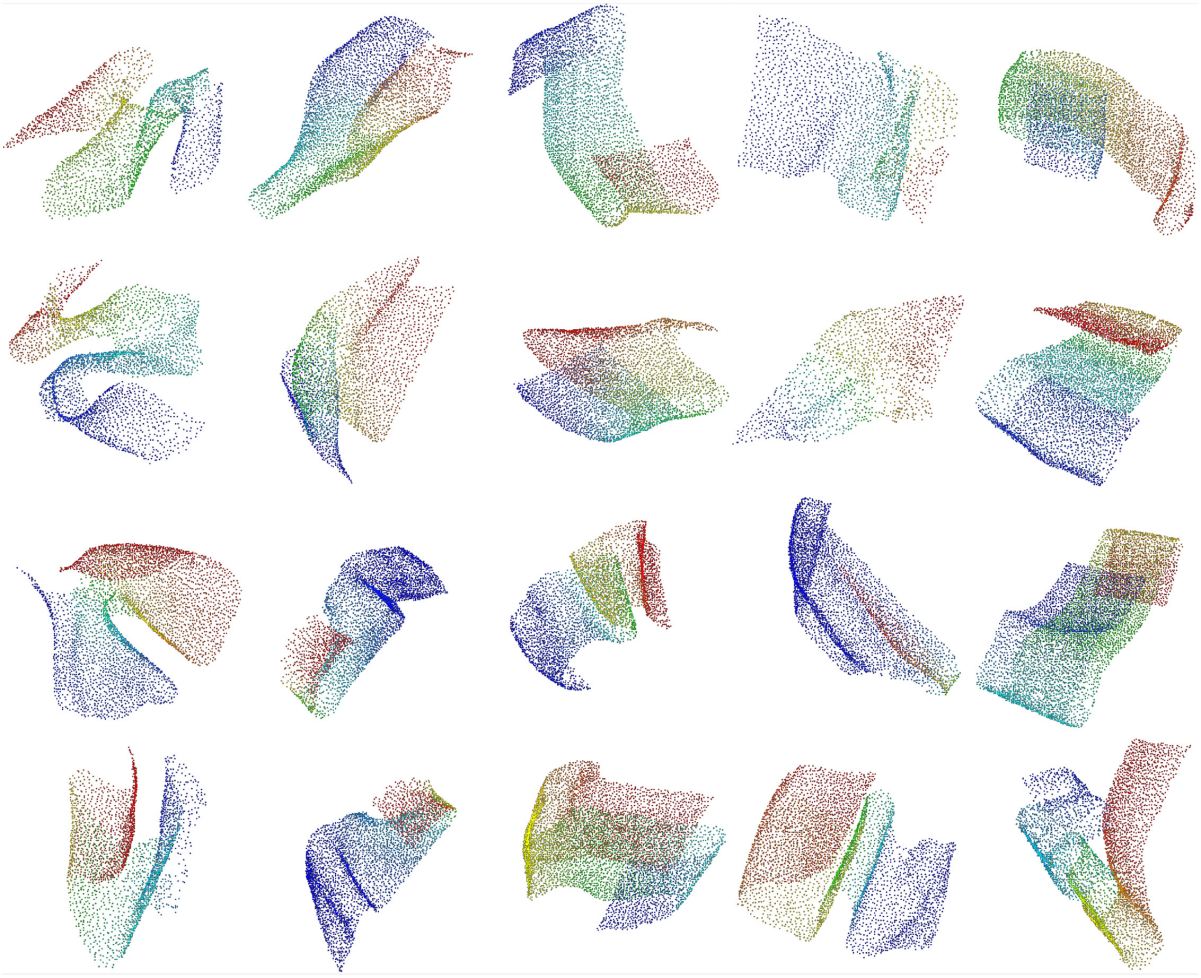
**Extracted charts from the Purkinje layer**. Examples of manually cleaned charts. Pseudo-colors (based on Fiedler vectors) are associated with points representing Purkinje somata in order to suggest their 3D arrangement.

We employed a semi-automatic approach to identify regions in the Purkinje layer where the spatial distribution of cell somata shows rarefactions. We started from the set of automatically detected soma centers as described above, but omitting the last processing stage to avoid as many false negatives as possible. Furthermore, we set the r parameter in the above procedure to 3 in order to achieve a recall as high as 0.98 measured on the 56 ground truth substacks. The point cloud was split into 15 overlapping slices with cuts perpendicular to the sagittal plane (each slice was about 2000 voxels high, with an overlap of 120 voxels). Each slice was manually cleaned up using the CloudCompare software^2^. In particular we removed obvious false positives (i.e., somata excessively delaminated with respect to the Purkinje layer), resulting in a set of 221107 soma centers for the whole cerebellum. Finally, from all the slices, we manually cut a total of 89 charts containing between 292 and 6023 soma centers each. We cut each slice along regions of minimal curvature and keeping a reasonable overlap between adjacent charts to prevent border effects in the subsequent analysis. **Figure 3** shows some examples of the extracted charts, each corresponding to a portion of the PC layer.

**FIGURE 4:**
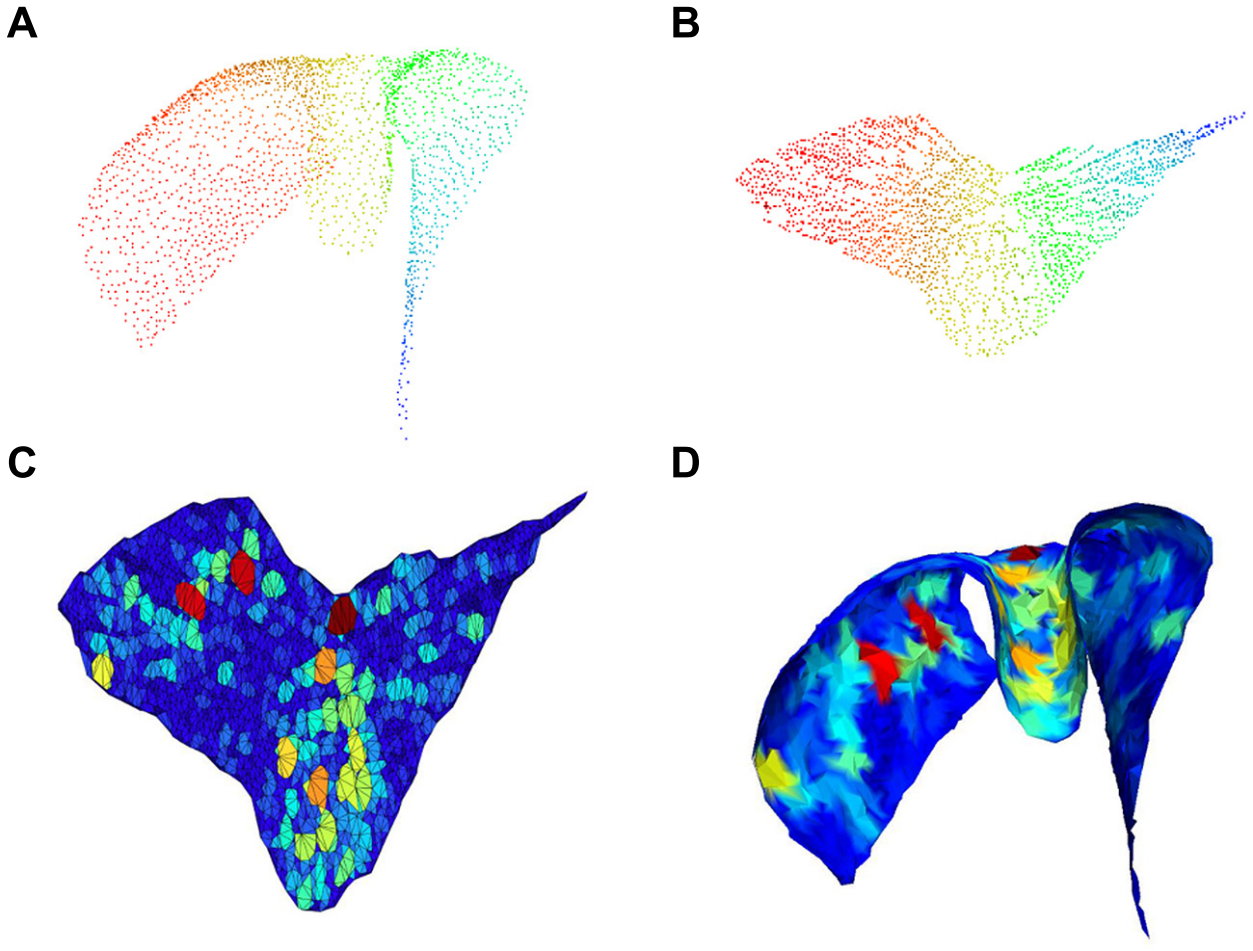
**Gap size estimation**. 3D cloud point of a single chart **(A)** and associated 2D embedding **(B)**; pseudo-colors (from Fiedler vector) are only used to identify points. Triangulation with colors proportional to void area (red highest) in the 2D embedding space is shown in **(C)** and corresponding mesh back in the 3D space in **(D)**.

Each chart was then automatically analyzed to identify the presence of gaps in the cell layout. We define gaps as large convex areas in the layer containing no cells. Finding holes or void regions in point clouds has been investigated in various forms in the literature. For example (Boyce et al., 1985) studied the problems of finding maximum perimeter and maximum area convex k-gons for given k. Unlike those previous studies, here we formally define the problem as follows: given a point in the surface described by the cell layer, we want to determine the largest surface portion containing that point and no cells. As a first step, our algorithm uses Isomap to embed 3D coordinates *(x, y, z)* of soma centers into the 2D space, with coordinates *(u, v)*, corresponding to the manifold describing a Purkinje layer folium. We subsequently seek convex polygons of maximal area in the 2D space. For this purpose, we first compute a Delaunay triangulation of the set of soma centers in the 2D space. Since the triangulation can produce artifacts, i.e., very large triangles connecting distant points, we delete them with an iterative approach, discarding at each iteration boundary triangles with perimeter above a threshold. For every triangle in the triangulation, we finally grow a region by adding adjacent triangles if the polygon resulting from an addition remains convex. Clearly, the convex regions created in this way do not contain any cell in their interiors. At the end, the algorithm returns, for every triangle, the area of the convex region grown around it. The steps described in this paragraph are illustrated in **Figure 4**.

The software used for gap localization can be downloaded from https://bitbucket.org/marco_paciscopi/manifold-find-holes. Developers can found extensive documentation on the same website.

### Image Visualization and Further Analysis

3D volume renderings of the original microscopy images were obtained with VAA3D^3^; meshes representing the layer folia were visualized with MeshLab^4^, while point clouds representing PCs were visualized using CloudCompare. Statistical analysis of the distribution of inter-cellular areas was performed using Matlab R2014b (MathWorks Inc., USA).

## Results

All the results shown below, as well as the raw image data, are available for download at https://dataverse.harvard.edu/dataverse/mouse_cerebellum

### Clustering of Purkinje Cells

**FIGURE 5:**
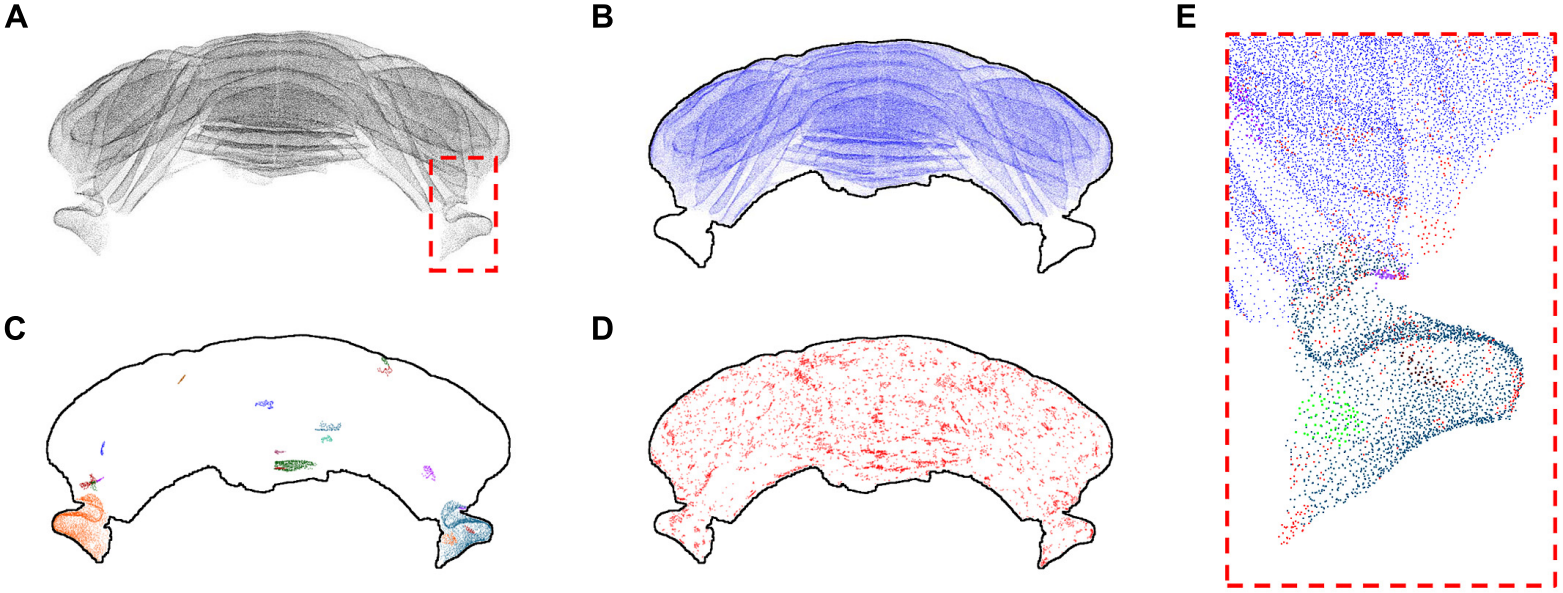
**k-Nearest neighbor clustering of Purkinje cells (*k* = 3)**. The whole Purkinje cells point cloud **(A)** and the largest cluster of kNN-connected cells **(B)**. The 20 biggest clusters after the first are shown with different colors in **(C)**, while in **(D)** all the remaining clusters and isolated cells are shown in red. The dashed red square depicted in **(A)** is shown at higher magnification in **(E)**, using a similar color scheme as the one used in **(B–D)**. All results for *k* = 3. In **(B–D)** a profile of the cerebellum is added to help the reader.

The refined version of the point cloud obtained with additional manual removal of false positives was processed according to the clustering algorithm discussed above (**Figure 5**). Setting the number *k* of nearest neighbors equal to 3, we found that most identified PCs (207190 out of 221107, almost 94%) cluster in a big class spanning the whole cerebellum, with the exclusion of the two side lobes (**Figure 5B**). The rest of the neurons, with the exception of two bigger clusters located in the lobes, is divided in small groups (**Figures 5C,D**). 1131 clusters are made by less than 100 cells, and 1389 isolated neurons are found. Smaller clusters and isolated cells seem to be distributed almost uniformly across the whole sample, although preferentially on the external regions of the cerebellum. If bigger values of *k* are used, the biggest component becomes the only significant one: for *k* = 5, almost 99.9% of PCs belong to this component, and the number of smaller clusters and of isolated neurons are reduced to 41 and 204, respectively. For *k* = 8, no isolated cells are found, and more than 99.9% of cells belong the biggest component.

The kNN clustering of the cerebellum seems thus to be more effective when the number of nearest neighbors considered is small. In this regime, clustering is more selective and smaller classes are preserved. For bigger *k* almost all PCs are clustered in a single component, highlighting their compact spatial organization. The parcellation of the sample we obtained using kNN clustering with *k* = 3 does not have a straightforward interpretation in terms of traditional neuroanatomy or physiology (i.e., being in the same cluster is unrelated to structural and functional connectivity). However, it could be a fine anatomical signature useful to compare subjects of different ages or in presence of a disease.

### Distributions of Gaps in the Purkinje Cells Layer

**FIGURE 6:**
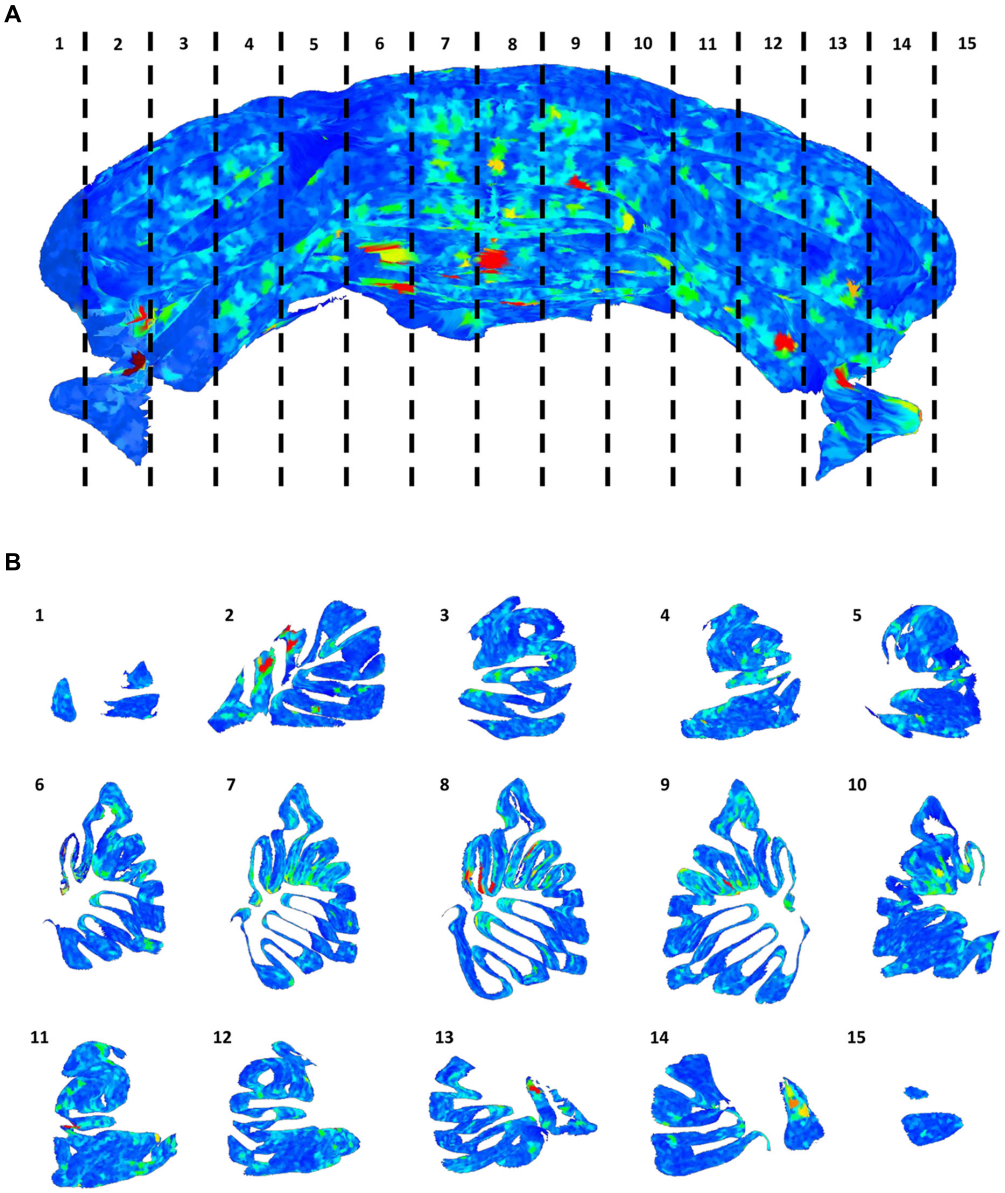
**Distribution of gaps in the Purkinje cells layer**. The full 3D mesh representing the Purkinje layer **(A)**, colored according to the size of empty areas between the cells (blue smallest, red largest). To help visualize the internal structure of the mesh, single slices (according to the numeration in **A**) are reported **(B)**.

**FIGURE 7:**
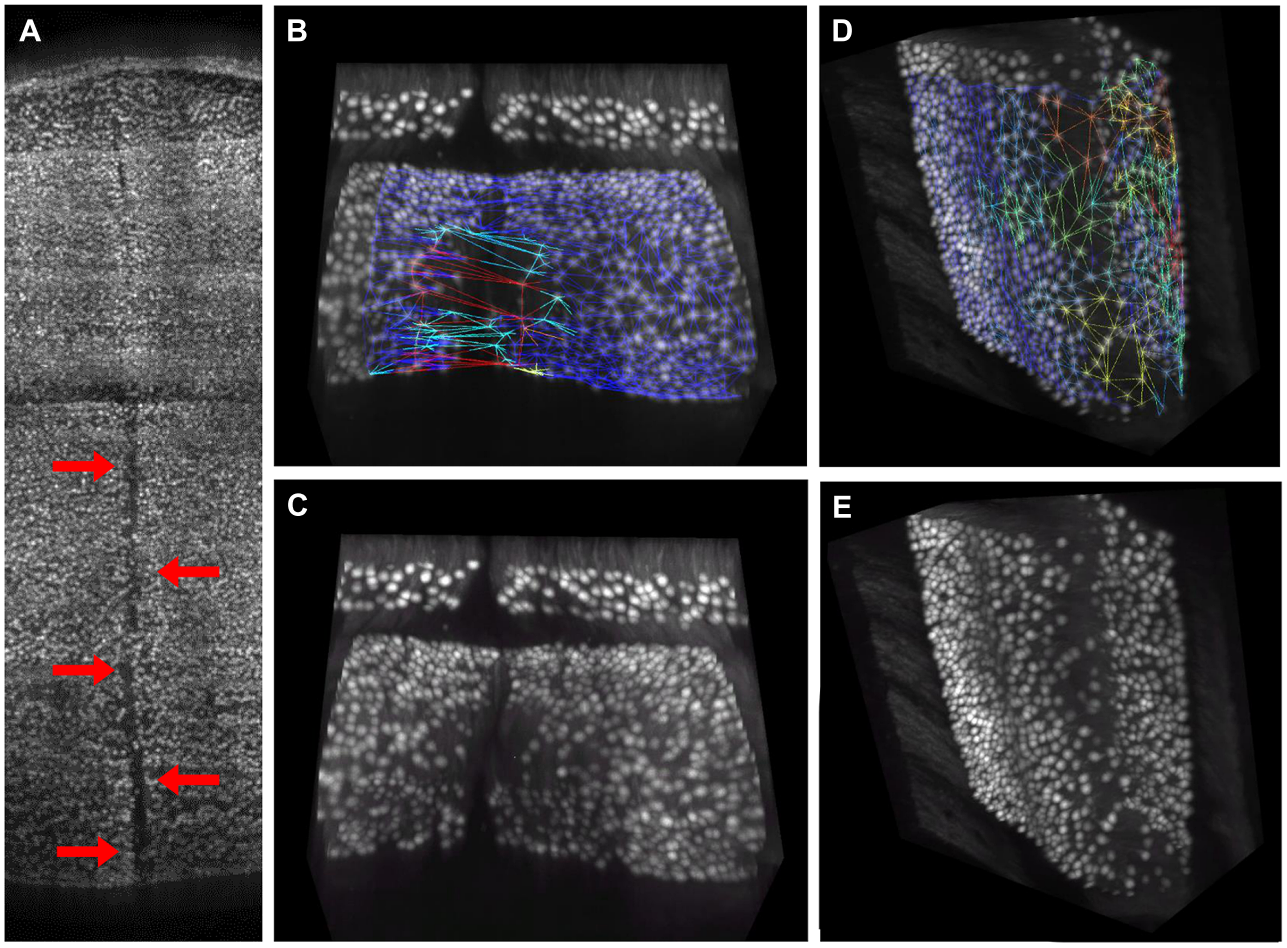
**Visual confirmation of gaps distribution map**. 3D rendering of the central region of the cerebellum, where a crack caused by tissue clearing (indicated by red arrows) is clearly visible **(A)**. This crack gives rise to large “pseudo-gaps” in the 3D mesh: a 3D rendering of a smaller volume with the mesh superimposed is reported in **(B)**. The same volume without mesh is in **(C)**. In absence of macroscopic cracks, the triangular mesh correctly highlights regions with smaller planar density of Purkinje cells, which are commonly found at the folium curvature: a representative volume with and without mesh is reported in **(D,E)**, respectively. Mesh triangles are colored according to their areas (blue smallest, red largest).

The distribution of gaps in the PC layer can be inferred by the presence of large convex polygons in the 2D manifolds locally describing the layer folia. In **Figure 6A**, a complete view of the cerebellum is shown where all these polygons are remapped to the 3D space and colored according to their area. If the whole dataset is sliced along the medio-lateral axis, the distribution of gaps in the complete lamellar structure becomes more evident (**Figure 6B**). In the Figure, three areas can be identified where inter-cellular distances are particularly large (in red): one in the middle and two close to the side lobes. However, from visual inspection of the original data, it turns out that in these regions the sample was partially cracked during clearing (**Figure 7A**). The large intercellular distance in these areas is therefore due to some artifact during tissue preparation, and not to the real distribution of gaps in the layer (**Figures 7B,C**).

On the other hand, from closer analysis of single slices it appears that (real) larger inter-cellular gaps are mostly localized in correspondence to the internal curvatures of the lamellar structure (**Figure 6B**). This is confirmed by visual inspection of the original data (**Figures 7D,E**). Considering the young age of the mouse (PND 10), this could be due to the incomplete migration or topographically localized apoptotic death of the PCs (Dusart et al., 2006; Castagna et al., 2014).

**FIGURE 8:**
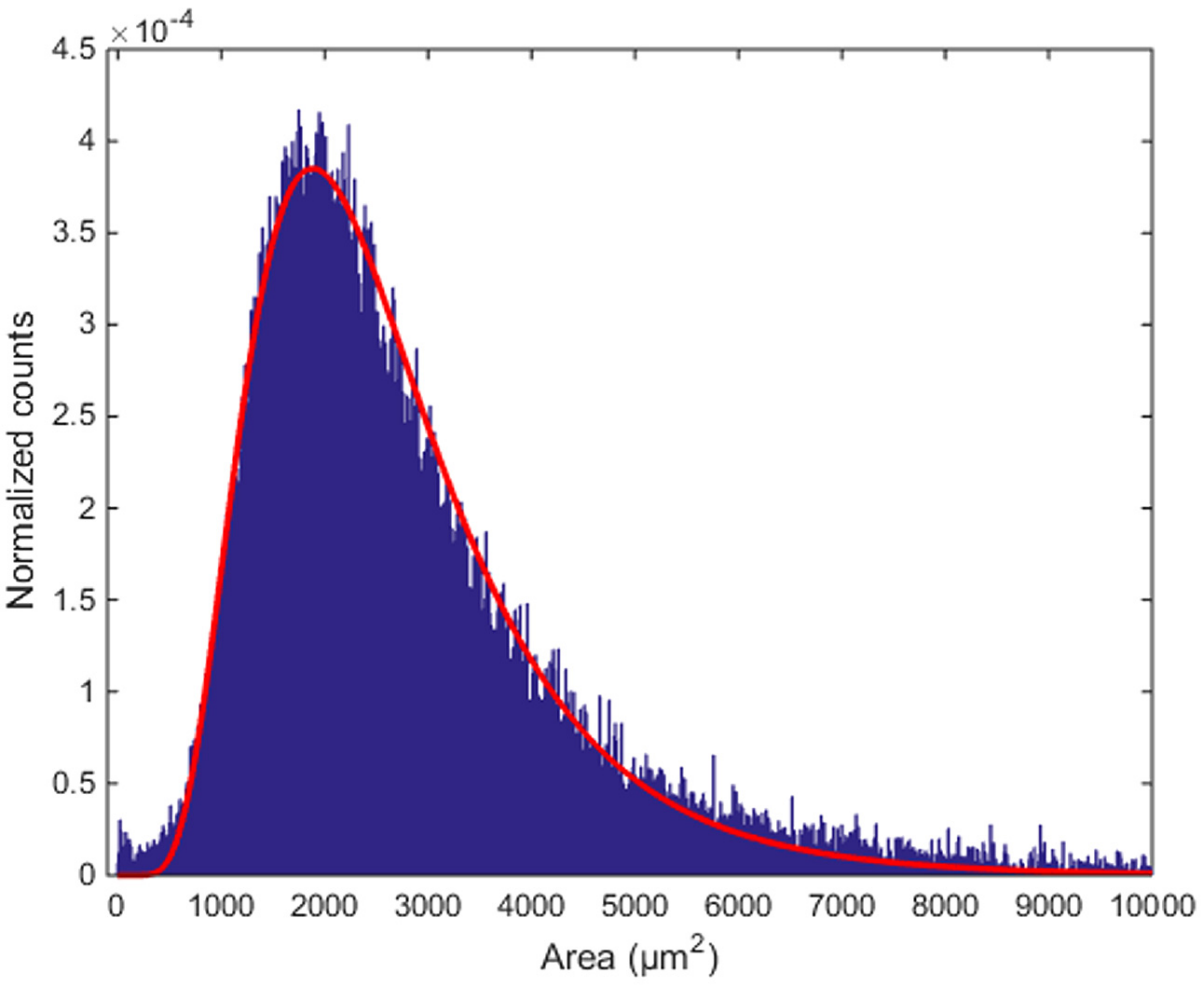
**Distribution of inter-cellular areas**. The histogram of empty areas in the Purkinje layer (in blue) and its fit with a log-normal distribution (in red).

The area of the largest convex polygon tangent to each cell show a mono-modal distribution, with mean value 2950 μm^2^ and standard deviation 2583 μm^2^ (**Figure 8**). This distribution can be well-fitted by a log-normal curve, with μ = 7.779 ±0.003 and σ = 0.488 ±0.002 (99% confidence intervals). This kind of probability distributions is quite common in neuroscience (Buzsaki and Mizuseki, 2014). Thus, although many different measurements from different animals would be needed to validate it, the findings that inter-cellular areas between PCs are distributed in a log-normal fashion seems at least plausible. In perspective, the distribution parameters can also be used as global fingerprints to compare different mice.

## Discussion

Contemporary neuroscience is in urgent need of a new generation of neuroanatomical techniques allowing scalable, reliable, specific, and quantitative analysis of macroscopic portions of brain tissue with cellular or sub-cellular resolution. Such a technical advance requires the integration of recent efforts in terms of transgenic animal development, sample clearing and staining, high-throughput imaging, and image analysis. Here, we presented a proof-of-principles of such combined approach on the cerebellum of an L7-GFP mouse. The sample was cleared with organic solvents and imaged with a confocal LSM; raw images produced by the apparatus were stitched together and subsequently analyzed to localize all PCs. Starting from the cloud of points representing all the Purkinje neurons in the cerebellum, further analysis was performed, highlighting both the clusterization properties of the point cloud and the distribution of gaps in the layer. Although we showed this experimental pipeline on a single sample, all the methods used (with the exception of cell clustering and gap localization) have been already demonstrated elsewhere (Becker et al., 2012; Silvestri et al., 2012; Frasconi et al., 2014), and a further validation of their capabilities is out of the scope of this work. The growing amount of papers exploiting sample clearing, LSM and the software tools described here, e.g. (Jahrling et al., 2010; Mertz and Kim, 2010; Erturk et al., 2012; Silvestri et al., 2014; Tomer et al., 2014), demonstrates the reliability of each single component of our workflow, paving the way to its application on a larger number of samples.

When repeated on a significantly cohort of mice, the measurements shown here can provide robust and bias-free insights into the distribution of PCs under different physiological or pathological conditions. For instance PCs loss can be quantified in heterozygous reeler (rl/+) mice, an animal model that has been used for studying the interplay of reelin deficiency with environmental factors during early development (Biamonte et al., 2014). Indeed, it has been reported that adult male rl/+ mice have reduced numbers of PCs, in comparison to female rl/+ mice and wild-type mice of either sex (Biamonte et al., 2009). The pipeline described in this paper can shine a new light in previous findings, allowing a more comprehensive characterization of the effect of reelin deficiency not only on the average number of PCs, but also on their spatial arrangement. The non-biased global analysis presented here can easily reveal if the reduction in the number of PCs reported previously is due to cell death or rather to ectopic cell migration.

Although our proof-of-principle was on a single specific cell population (Purkinje neurons) and in a portion of the mouse brain (the cerebellum), the integrative approach we describe can be extended to different animal models (highlighting other cell types) and to larger specimens (as whole murine brains). In fact, recent advances in tissue clearing, as CLARITY (Chung et al., 2013) or CUBIC (Susaki et al., 2014), and in LSM (Tomer et al., 2014) are leading to a next generation of imaging protocols capable of producing high-resolution and high-contrast reconstructions of cm-wide samples. Furthermore, the ability of CLARITY of immunostaining macroscopic tissue portions can pave the way for quantitative large-volume neuroanatomy in humans as well as in non-human primates.

High-throughput imaging methods with improved contrast and resolution would both benefit and challenge computational tools. On the one hand, better images will increase the robustness and reliability of software: image stitching will be more precise since the cross-correlation will have a sharper peak, and cell localization will be more accurate because of the improved signal-to-noise ratio. On the other hand, the size of data is going to go well-beyond the TeraByte threshold, as soon as one is able to collect high-quality data from larger samples. Therefore, existing software tools should be adapted to cope with larger datasets, exploiting parallel architectures for both data processing and storage.

Beyond improving single technical aspects, as microscopy or image analysis, special efforts should be devoted to the integration of all those methodologies in a common and well-coordinated experimental pipeline. In this respect, it is crucial to have visualization and annotation tools designed for large images (Peng et al., 2014) that can be used to tune the protocols and for quality check. For instance, the comparison with original data was crucial to interpret the results found here, in particular to identify artifacts due to specimen cracking. Furthermore, since large-scale quantitative neuroanatomy is still in its infancy, a lot of trial and error will be needed to find out the best analysis methods and to properly use and interpret them. As an example, removal of localized points too far from the Purkinje layer significantly improves the quality of results (Frasconi et al., 2014), but may lead to biased analysis when cells are located quite out of the specimen – for instance in very early development stages (Larouche et al., 2008; Miyata et al., 2010). Manual inspection of images is also recommended to check the consistency of image stitching, which might fail when image quality is very low (Bria and Iannello, 2012).

## Conclusion

We demonstrated that when state-of-the-art methodologies from various fields are properly combined they can produce data that would have been out of the neuroanatomists’ reach only a few years ago. If this integrated approach will be kept updated with the most recent advances in each field, in the next decades researchers can gain further and further insight into the complexity of brain anatomy, chasing the ultimate dreams of Golgi and Ramòn y Cajal.

## Conflict of Interest Statement

The authors declare that the research was conducted in the absence of any commercial or financial relationships that could be construed as a potential conflict of interest.

## Footnotes

1 Recall, precision and F_1_ measure are standard performance measures in information retrieval. Recall (*R*) is defined as $\frac{TP}{TP+FN}$, Precision (*P*) is defined as $\frac{TP}{TP+FP},\;and\,\;F_{1}=\frac{2PR}{P+R}$. TP means True Positives, FP False Positives, FN False Negatives.

2 http://www.danielgm.net/cc/, Open Source Project managed by Daniel Girardeau-Monteaut.

3 http://home.penglab.com/proj/vaa3d/Vaa3D/About_Vaa3D.html

4 http://meshlab.sourceforge.net/

## References

[B1] Becker K. Jahrling N. Saghafi S. Weiler R. Dodt H. U. (2012). Chemical clearing and dehydration of GFP expressing mouse brains. *PLoS ONE* 7:e33916 10.1371/journal.pone.0033916 PMC3316521 22479475

[B2] Biamonte F. Assenza G. Marino R. D’Amelio M. Panteri R. Caruso D. (2009). Interactions between neuroactive steroids and reelin haploinsufficiency in Purkinje cell survival. *Neurobiol. Dis.* 36 103–115. 10.1016/j.nbd.2009.07.001 19595767

[B3] Biamonte F. Latini L. Giorgi F. S. Zingariello M. Marino R. De Luca R. (2014). Associations among exposure to methylmercury, reduced Reelin expression, and gender in the cerebellum of developing mice. *Neurotoxicology* 45 67–80. 10.1016/j.neuro.2014.09.006 25305366

[B4] Boyce J. E. Dobkin D. P. Drysdale R. L. Guibas L. J. (1985). Finding extremal polygons. *SIAM J. Comput.* 14 134–147. 10.1137/0214011

[B5] Bria A. Iannello G. (2012). TeraStitcher – a tool for fast automatic 3D-stitching of teravoxel-sized microscopy images. *BMC Bioinformatics* 13:316 10.1186/1471-2105-13-316 PMC3582611 23181553

[B6] Briggman K. L. Denk W. (2006). Towards neural circuit reconstruction with volume electron microscopy techniques. *Curr. Opin. Neurobiol.* 16 562–570. 10.1016/j.conb.2006.08.010 16962767

[B7] Briggman K. L. Helmstaedter M. Denk W. (2011). Wiring specificity in the direction-selectivity circuit of the retina. *Nature* 471 183–188. 10.1038/nature09818 21390125

[B8] Buzsaki G. Mizuseki K. (2014). The log-dynamic brain: how skewed distributions affect network operations. *Nat. Rev. Neurosci.* 15 264–278. 10.1038/nrn3687 24569488 PMC4051294

[B9] Castagna C. Aimar P. Alasia S. Lossi L. (2014). Post-natal development of the Reeler mouse cerebellum: an ultrastructural study. *Ann. Anat.* 196 224–235. 10.1016/j.aanat.2013.11.004 24411683

[B10] Chung K. Wallace J. Kim S. Y. Kalyanasundaram S. Andalman A. S. Davidson T. J. (2013). Structural and molecular interrogation of intact biological systems. *Nature* 497 332–337. 10.1038/nature12107 23575631 PMC4092167

[B11] Cleveland W. S. Devlin S. J. (1988). Locally weighted regression – an approach to regression-analysis by local fitting. *J. Am. Stat. Assoc.* 83 596–610. 10.1080/01621459.1988.10478639

[B12] Comaniciu D. Meer P. (2002). Mean shift: a robust approach toward feature space analysis. *IEEE Trans. Pattern Anal. Mach. Intell.* 24 603–619. 10.1109/34.1000236

[B13] Cover T. Hart P. (1967). Nearest neighbor pattern classification. *IEEE Trans. Inf. Theory* 13 21–27. 10.1109/TIT.1967.1053964

[B14] Dodt H. U. Leischner U. Schierloh A. Jahrling N. Mauch C. P. Deininger K. (2007). Ultramicroscopy: three-dimensional visualization of neuronal networks in the whole mouse brain. *Nat. Methods* 4 331–336. 10.1038/nmeth1036 17384643

[B15] Dusart I. Guenet J. L. Sotelo C. (2006). Purkinje cell death: differences between developmental cell death and neurodegenerative death in mutant mice. *Cerebellum* 5 163–173. 10.1080/14734220600699373 16818391

[B16] Duyn J. Koretsky A. P. (2008). Magnetic resonance imaging of neural circuits. *Nat. Clin. Pract. Cardiovasc. Med.* (5 Suppl. 2), S71–S78. 10.1038/ncpcardio1248 18641610 PMC3529508

[B17] Erturk A. Mauch C. P. Hellal F. Forstner F. Keck T. Becker K. (2012). Three-dimensional imaging of the unsectioned adult spinal cord to assess axon regeneration and glial responses after injury. *Nat. Med.* 18 166–171. 10.1038/nm.2600 22198277

[B18] Frasconi P. Silvestri L. Soda P. Cortini R. Pavone F. S. Iannello G. (2014). Large-scale automated identification of mouse brain cells in confocal light sheet microscopy images. *Bioinformatics* 30 i587–i593. 10.1093/bioinformatics/btu469 25161251 PMC4147922

[B19] Gong H. Zeng S. Yan C. Lv X. Yang Z. Xu T. (2013). Continuously tracing brain-wide long-distance axonal projections in mice at a one-micron voxel resolution. *Neuroimage* 74 87–98. 10.1016/j.neuroimage.2013.02.005 23416252

[B20] Gray E. G. (1969). Electron microscopy of excitatory and inhibitory synapses: a brief review. *Prog. Brain Res.* 31 141–155. 10.1016/S0079-6123(08)63235-5 4899407

[B21] Jahrling N. Becker K. Schonbauer C. Schnorrer F. Dodt H. U. (2010). Three-dimensional reconstruction and segmentation of intact *Drosophila* by ultramicroscopy. *Front. Syst. Neurosci.* 4:1 10.3389/neuro.06.001.2010 PMC2831709 20204156

[B22] Keller A. L. Zeidler D. Kemen T. (2014). High throughput data acquisition with a multi-beam SEM. *Scanning Microsc.* 2014:9236.

[B23] Keller P. J. Dodt H. U. (2012). Light sheet microscopy of living or cleared specimens. *Curr. Opin. Neurobiol.* 22 138–143. 10.1016/j.conb.2011.08.003 21925871

[B24] Keller P. J. Schmidt A. D. Wittbrodt J. Stelzer E. H. (2008). Reconstruction of zebrafish early embryonic development by scanned light sheet microscopy. *Science* 322 1065–1069. 10.1126/science.1162493 18845710

[B25] Kim J. S. Greene M. J. Zlateski A. Lee K. Richardson M. Turaga S. C. (2014). Space-time wiring specificity supports direction selectivity in the retina. *Nature* 509 331–336. 10.1038/nature13240 24805243 PMC4074887

[B26] Knott G. Marchman H. Wall D. Lich B. (2008). Serial section scanning electron microscopy of adult brain tissue using focused ion beam milling. *J. Neurosci.* 28 2959–2964. 10.1523/JNEUROSCI.3189-07.2008 18353998 PMC6670719

[B27] Larouche M. Beffert U. Herz J. Hawkes R. (2008). The Reelin receptors Apoer2 and Vldlr coordinate the patterning of Purkinje cell topography in the developing mouse cerebellum. *PLoS ONE* 3:e1653 10.1371/journal.pone.0001653 PMC2242849 18301736

[B28] Li A. Gong H. Zhang B. Wang Q. Yan C. Wu J. (2010). Micro-optical sectioning tomography to obtain a high-resolution atlas of the mouse brain. *Science* 330 1404–1408. 10.1126/science.1191776 21051596

[B29] Lichtman J. W. Conchello J. A. (2005). Fluorescence microscopy. *Nat. Methods* 2 910–919. 10.1038/nmeth817 16299476

[B30] McKimm E. Corkill B. Goldowitz D. Albritton L. M. Homayouni R. Blaha C. D. (2014). Glutamate dysfunction associated with developmental cerebellar damage: relevance to autism spectrum disorders. *Cerebellum* 13 346–353. 10.1007/s12311-013-0541-4 24307139 PMC4060592

[B31] Meinertzhagen I. A. Takemura S. Y. Lu Z. Huang S. Gao S. Ting C. Y. (2009). From form to function: the ways to know a neuron. *J. Neurogenet.* 23 68–77. 10.1080/01677060802610604 19132600

[B32] Mertz J. Kim J. (2010). Scanning light-sheet microscopy in the whole mouse brain with HiLo background rejection. *J. Biomed. Opt.* 15:016027 10.1117/1.3324890 PMC2917465 20210471

[B33] Miyata T. Ono Y. Okamoto M. Masaoka M. Sakakibara A. Kawaguchi A. (2010). Migration, early axonogenesis, and Reelin-dependent layer-forming behavior of early/posterior-born Purkinje cells in the developing mouse lateral cerebellum. *Neural Dev.* 5:23 10.1186/1749-8104-5-23 PMC2942860 20809939

[B34] Oberdick J. Smeyne R. J. Mann J. R. Zackson S. Morgan J. I. (1990). A promoter that drives transgene expression in cerebellar Purkinje and retinal bipolar neurons. *Science* 248 223–226. 10.1126/science.2109351 2109351

[B35] Osten P. Margrie T. W. (2013). Mapping brain circuitry with a light microscope. *Nat. Methods* 10 515–523. 10.1038/nmeth.2477 23722211 PMC3982327

[B36] Palay S. L. (1956). Synapses in the central nervous system. *J. Biophys. Biochem. Cytol.* 2 193–202. 10.1083/jcb.2.4.193 13357542 PMC2229686

[B37] Peng H. Ruan Z. Long F. Simpson J. H. Myers E. W. (2010). V3D enables real-time 3D visualization and quantitative analysis of large-scale biological image data sets. *Nat. Biotechnol.* 28 348–353. 10.1038/nbt.1612 20231818 PMC2857929

[B38] Peng H. Tang J. Xiao H. Bria A. Zhou J. Butler V. (2014). Virtual finger boosts three-dimensional imaging and microsurgery as well as terabyte volume image visualization and analysis. *Nat. Commun.* 5:4342 10.1038/ncomms5342 PMC4104457 25014658

[B39] Ragan T. Kadiri L. R. Venkataraju K. U. Bahlmann K. Sutin J. Taranda J. (2012). Serial two-photon tomography for automated ex vivo mouse brain imaging. *Nat. Methods* 9 255–258. 10.1038/nmeth.1854 22245809 PMC3297424

[B40] Rockland K. S. (2010). Five points on columns. *Front. Neuroanat.* 4:22 10.3389/fnana.2010.00022 PMC2893004 20589097

[B41] Sahoo P. K. Soltani S. Wong A. K. C. Chen Y. C. (1988). A survey of thresholding techniques. *Comput. Vis. Graph. Image Process.* 41 233–260. 10.1016/0734-189X(88)90022-9

[B42] Silvestri L. Allegra Mascaro A. L. Costantini I. Sacconi L. Pavone F. S. (2014). Correlative two-photon and light sheet microscopy. *Methods* 66 268–272. 10.1016/j.ymeth.2013.06.013 23806642

[B43] Silvestri L. Bria A. Sacconi L. Iannello G. Pavone F. S. (2012). Confocal light sheet microscopy: micron-scale neuroanatomy of the entire mouse brain. *Opt. Express* 20 20582–20598. 10.1364/OE.20.020582 23037106

[B44] Susaki E. A. Tainaka K. Perrin D. Kishino F. Tawara T. Watanabe T. M. (2014). Whole-brain imaging with single-cell resolution using chemical cocktails and computational analysis. *Cell* 157 726–739. 10.1016/j.cell.2014.03.042 24746791

[B45] Tench C. R. Morgan P. S. Wilson M. Blumhardt L. D. (2002). White matter mapping using diffusion tensor MRI. *Magn. Reson. Med.* 47 967–972. 10.1002/mrm.10144 11979576

[B46] Tenenbaum J. B. De Silva V. Langford J. C. (2000). A global geometric framework for nonlinear dimensionality reduction. *Science* 290 2319–2323. 10.1126/science.290.5500.2319 11125149

[B47] Tomer R. Ye L. Hsueh B. Deisseroth K. (2014). Advanced CLARITY for rapid and high-resolution imaging of intact tissues. *Nat. Protoc.* 9 1682–1697. 10.1038/nprot.2014.123 24945384 PMC4096681

